# Automatic Supporting System for Regionalization of Ventricular Tachycardia Exit Site in Implantable Defibrillators

**DOI:** 10.1371/journal.pone.0124514

**Published:** 2015-04-24

**Authors:** Margarita Sanromán-Junquera, Inmaculada Mora-Jiménez, Jesús Almendral, Arcadio García-Alberola, José Luis Rojo-Álvarez

**Affiliations:** 1 Department of Signal Theory and Communications, Telematics and Computing, Universidad Rey Juan Carlos, Fuenlabrada 28943, Spain; 2 Arrhythmia Unit, Hospital Madrid Montepríncipe, Boadilla del Monte 28660, Spain; 3 Arrhythmia Unit, Hospital Universitario Virgen de la Arrixaca, Murcia 30120, Spain; 4 Universidad de las Fuerzas Armadas-ESPE, Sangolquí 171-5-231B, Ecuador; University of Minnesota, UNITED STATES

## Abstract

Electrograms stored in Implantable Cardioverter Defibrillators (ICD-EGM) have been proven to convey useful information for roughly determining the anatomical location of the Left Ventricular Tachycardia exit site (LVTES). Our aim here was to evaluate the possibilities from a machine learning system intended to provide an estimation of the LVTES anatomical region with the use of ICD-EGM in the situation where 12-lead electrocardiogram of ventricular tachycardia are not available. Several machine learning techniques were specifically designed and benchmarked, both from classification (such as Neural Networks (NN), and Support Vector Machines (SVM)) and regression (Kernel Ridge Regression) problem statements. Classifiers were evaluated by using accuracy rates for LVTES identification in a controlled number of anatomical regions, and the regression approach quality was studied in terms of the spatial resolution. We analyzed the ICD-EGM of 23 patients (18±10 EGM per patient) during left ventricular pacing and simultaneous recording of the spatial coordinates of the pacing electrode with a navigation system. Several feature sets extracted from ICD-EGM (consisting of times and voltages) were shown to convey more discriminative information than the raw waveform. Among classifiers, the SVM performed slightly better than NN. In accordance with previous clinical works, the average spatial resolution for the LVTES was about 3 cm, as in our system, which allows it to support the faster determination of the LVTES in ablation procedures. The proposed approach also provides with a framework suitable for driving the design of improved performance future systems.

## Introduction

Ventricular Tachycardia (VT) and Ventricular Fibrillation (VF) are two of the most relevant causes of sudden cardiac death. A widely used therapy for arrhythmias is the Implantable Cardioverter Defibrillator (ICD), a life-support device which automatically detects the presence of life-threatening heart rhythms and subsequently delivers an appropriate therapy, such as controlled electrical stimulation or shock [1]. Another therapy for ventricular arrhythmias is radiofrequency ablation in electrophysiological studies (EPS), which has been proven to be one of the most effective procedures to reduce the incidence of arrhythmic events.

Most of VTs are due to slow conduction pathways within cardiac scar tissue in patients with structural heart disease. During VT ablation therapy, the knowledge of the point where the healthy myocardial tissue is activated by the slow conduction pathway can be very useful to ensure the therapy success. This point is often known as *exit site*, and one technique for its identification is the pace mapping procedure, consisting of reproducing the beat morphology recorded during VT by applying short bursts of controlled and regular electrical stimuli at different parts of the ventricle. By using a mapping catheter for this purpose, the exit site is found when the beat morphology obtained during pace mapping is very similar to the beat morphology of the clinically observed VT in terms of the 12 lead electrocardiogram (ECG). However, patients with ICD do not usually have their ECG recorded during out-of-hospital VT episodes, and the only electrophysiological information available in these patients comes from the intracardiac electrograms (EGM) recorded by the ICD at that episode. Therefore, a study about the capability of the EGM stored in ICD for locating the VT exit site (VTES) could be very useful for providing the clinician with relevant information at the subsequent EPS, which could support and significantly reduce the time required for the cardiac ablation procedure.

Previous research in this setting has focussed on predicting the VTES based on 12-lead surface ECG [2, 3]. From an EPS point of view, scientific evidence supports the morphology of EGM from ICD providing relevant information about the region where the VTES comes from. In [4], the arrhythmia anatomical exit site location in the left ventricular (LV) anatomy (i.e. septal vs lateral, superior vs inferior, and apical vs basal) was determined by using features from the EGM waveform, such as the voltage of far-field EGM deflections (initial, peak, and final), as well as time intervals between far-field and bipolar EGM deflections. In that work, the ratio between initial and peak voltages in the far-field EGM was used to distinguish lateral vs septal, and the interval onset of far-field to bipolar EGM was used to distinguish LV halves (septal, lateral, apical, basal). Several studies have focused on identifying the spatial resolution of EGM [4–6]. In [5], the mean spatial resolution of 12-lead ECG and EGM pace-maps was compared for the determination of VTES, concluding that better spatial resolution was obtained with 12-lead ECG (2.9 ± 4.0 cm^2^) than with EGM (8.9 ± 9.0 cm^2^), regardless this result pointed out that EGM morphology might be useful to roughly identify the VTES. In [6], a case was presented, for which the 12-lead ECG did not provide enough precision for delimiting the VT ablation area, whereas EGM helped the clinicians to define a more reduced area for successful ablation of the VTES. The distance between pacing sites was shown in [4] to be higher than 2 cm in order to distinguish EGM registered in both sites.

Despite of previous evidence, no automatic system has been provided for supporting the anatomical region identification of the left ventricular tachycardia exit site (LVTES) in ablation procedures from the principled analysis of the EGM stored in the ICD during preceding spontaneous arrhythmias. Aforementioned studies [4–6] have shown that the EGM spatial resolution is about several centimeters, hence the anatomical region of the LVTES could be (at least roughly) identified by considering LV regions. Therefore, our aim in this work was to propose, test the general feasibility, and technically evaluate an operative and automatic machine learning system to support ablation procedures for the anatomical region identification of LVTES when the 12-lead ECG is not available and the only information is the EGM. For this purpose, both features (voltages and time) and EGM waveform registered by ICD during pacing in a pace-mapping protocol were analyzed, and conclusions of this analysis were extrapolated to the LVTES determination in spontaneous arrhythmias. In addition, a different machine learning system was scrutinized, in order to determine the EGM spatial resolution, i.e. the distance between the actual and the estimated LVTES, and our system performance was compared in detail with the expected accuracy that has been previously reported in the clinical literature.

Different approaches were used in this work to identify the region of arrhythmia exit site. We decided to use a supervised learning scheme, given that both EGM and corresponding pacing spatial position during pace mapping (pacing coordinates) were simultaneously available in our patient database. In order to tackle with the LV region identification, a manual division of the LV was done by following the methodology proposed in [4], and accounting for the LV anatomical sections used in the clinical practice. This division allowed us to tackle the identification in regions from a classification point of view (classes corresponded to regions of the divided LV), and representative machine learning classifiers were evaluated. In order to estimate the spatial resolution provided by the learning systems, a regression approach was used in terms of the actual spatial coordinates of each pacing site as target outputs. A very preliminary version of this work was presented in [7].

The paper is organized as follows. The following section presents the stages of the proposed supporting system, including the description of the considered machine learning techniques and the methods for evaluating their performance. Then, details are given about the patient database and the input features extraction. The next section describes several experiments for identifying the anatomical region of VTES using classification and regression machines. Experimental results are benchmarked by using both the raw EGM waveform and relevant sets of features as inputs to the machine learning algorithms. Finally, discussion and conclusions are summarized.

## Materials and Methods

### Ethics statement

The entire study was approved by Ethics Committee of the Hospital General Universitario Gregorio Marañón in Madrid (Spain). Written informed consent was obtained from all participants.

### Clinical Information and Database

The clinical information has been previously reported with detail in [4]. In brief, EGM were obtained from 23 patients evaluated in EPS for arrhythmia ablation therapy at the Hospital General Universitario Gregorio Marañón in Madrid (Spain). Patients included in the study had previously implanted a Medtronic™ICD, and a LV mapping procedure was performed for clinical reasons by using a 3D nonfluoroscopic Cardiac Navigation System (CNS). Patients (20 men and 3 women) were 69 ± 10 (mean±standard deviation) years old, and all of them had structural heart disease, namely, 18 post myocardial infraction, 3 idiopathic dilated cardiomyopathy, 1 bi-ventricular dysplasia, and 1 localized LV disynergy. All patients had documented previous VT, and the LV ejection fraction depression degree was estimated as mild (3), moderate (4), and severe (16 patients).

EGM were recorded by an ICD electrode located at the right ventricular apex and a left subclavicular can during fast LV pacing. Pacing protocol at each LV site consisted of trains of at least 10 beats of rapid LV bipolar pacing (cycle length of 500 or 400 milliseconds), in order to avoid both a worsening of the patients’ clinical situation, and inducing a number of VT being different to the clinical VT. Hence, the average length of the recorded signals was from 4 to 5 seconds. All paced beats from each site were visually analyzed to exclude sinus captures, fusion beats, and extrasystoles. EGM were simultaneously stored in the ICD by means of two different lead configurations, namely, far-field (can to coil) and bipolar (tip to ring). EGM and their corresponding octants were available for 23 patients, 18 ± 10 EGM per patient. In 11 out of 23 patients, EGM and spatial coordinates (20 ± 9 EGM per patient) were also available. In addition, the classification of pacing sites according to the peak-to-peak EGM voltage as normal (≥ 1.5 mV) or scar (< 1.5mV) was also provided with 12 out of 23 patients (15 ± 8 EGM per patient, 8 ± 6 EGM per patient in scar areas, and 7 ± 5 EGM per patient in normal areas). Note that this study was performed within clinical procedures of mapping and ablation, and therefore patients had a compromised cardiac health status. Since pacing and therapy had to focus as much as possible on the patient response, the distribution of pacing sites was not equally represented in all octants, as it is presented in Table 1, which shows with detail the number of EGM per octant.

**Table 1:** Number of EGM per octant in our database.

Octant	Number of EGM
Basal—Septal—Superior (BSS)	37
Basal—Septal—Inferior (BSI)	38
Basal—Lateral—Superior (BLS)	64
Basal—Lateral—Inferior (BLI)	94
Apical—Septal—Superior (ASS)	45
Apical—Septal—Inferior (ASI)	41
Apical—Lateral—Superior (ALS)	50
Apical—Lateral—Inferior (ALI)	46

Digital EGM were not available due to potential commercial interests. Therefore, the EGM were collected by telemetry from ICD during EPS and printed at a paper speed of 50 mm/s. Then, an algorithm to extract the EGM signal from printed-paper recordings was needed in order to have the EGM signals available in digital format. An algorithm for EGM digital recovery based on digital image processing techniques was used to recover the ICD-EGM from printout format. This method was carefully evaluated visually and quantitatively by comparing the gold-standard digital signal and the recovered signal with several merit figures, see [8, 9] for details.

The input space for the machine learning system was obtained both from the recordings of the can-coil and tip-ring lead configuration, corresponding to far-field and bipolar EGM, respectively. Firstly, a cubic smoothing spline filter was applied for EGM baseline removal. Then, a representative beat waveform template was obtained at every pacing site by averaging the available beats. Peak deflection of far-field EGM waveform was used to synchronize and to average the available beats. Note that there could be some small temporal variations in the waveform morphology during the pacing protocol due to small shift of the catheter because of the patients breath or the heart movements. However, the EGM was discarded when perceptible shift was detected by the electrophysiologist. Two datasets were built: (D1) the morphology of the averaged beat waveform, i.e. samples of the beat waveform template; and (D2) representative features obtained from the beat waveform template.

The averaged beat waveform of dataset D1 consisted of 150 voltage samples, with a total duration of 254 ms. In order to analyze the effect of the stimulus in the learning system, we defined two subsets for far-field EGM waveform (D1): the beat waveform template with the stimulus corresponding to the pace mapping stimulation, and the beat waveform template without the stimulus. The stimulus artifact was discarded by applying linear interpolation between previous and posterior samples to it. In addition, a new dataset was created by the concatenation of far-field and bipolar EGM waveforms, in order to evaluate whether the simultaneous consideration of both EGM provided complementary information.

**Fig 1:**
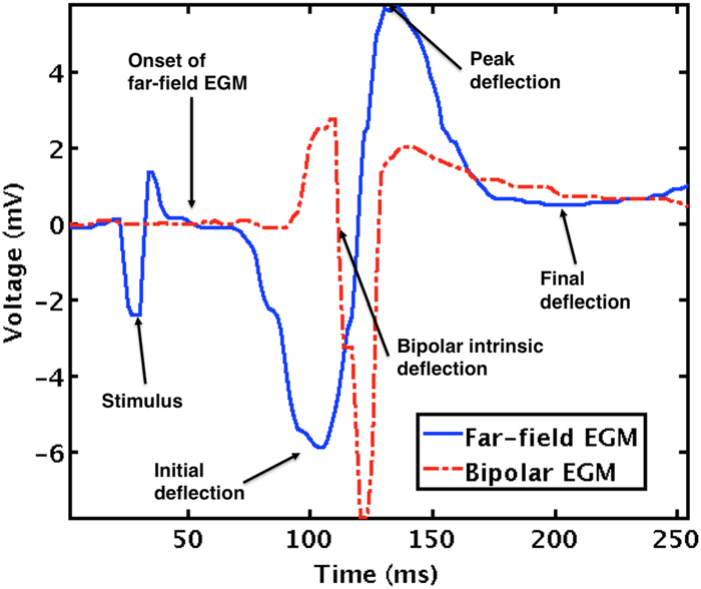
Example of a beat waveform. Representative features for far-field continuous line and bipolar EGM.

Dataset D2 considered several features of far-field and bipolar EGM (see Fig 1) that have been shown significant for helping in the regionalization of the impulse site formation within the LV endocardium [4]:

- **Voltages**: voltage of peak deflection (*v*_*P*_), which is the highest voltage above the baseline; voltage of initial deflection (*v*_*I*_), which is the smallest voltage between the onset of far-field EGM and the peak deflection (note that *v*_*I*_ is usually a negative value); voltage of final deflection (*v*_*F*_), which is the smallest voltage in an interval of 170 ms after the peak deflection instant.
- **Voltage ratios**: voltage ratio between final and peak deflections (*v*_*FP*_); voltage ratio between initial and peak deflections (*v*_*IP*_).
- **Time interval and instants**: *t*_*ob*_, or time interval between the onset of far-field EGM and the bipolar intrinsic deflection; *t*_*P*_, or time instant of *v*_*P*_; *t*_*I*_, or time instant of *v*_*I*_; *t*_*F*_, or time instant of *v*_*F*_. The time origin corresponded to the mid-time between previous and current peak deflections.

According to conclusions in [4], the following feature combinations were used in dataset D2:

- **3V**: *v*_*I*_, *v*_*P*_, and *v*_*F*_.
- **3V1T**: *v*_*I*_, *v*_*P*_, *v*_*F*_, and *t*_*ob*_.
- **3V3T**: *v*_*I*_, *v*_*P*_, *v*_*F*_, *t*_*I*_, *t*_*P*_, and *t*_*F*_.
- **2VR1T**: *v*_*FP*_, *v*_*IP*_, and *t*_*ob*_.

It was convenient to normalize every feature, in order to avoid dominant input attributes due to scaling during the learning procedure [10]. A zero mean and unit standard deviation normalization was used in our experiments.

### Proposed System

In this section, the theoretical framework is presented for a new data-driven system aimed to support ablation procedures by using EGM (waveform and features) recorded during EPS. Machine learning techniques are used both for system design and for operation. Emphasis is made in supervised learning and capabilities to extract information from the EGM in terms of the VT anatomical exit site. Additionally, a quality measurement stage is included in order to evaluate the statistical properties of the output provided by different machines. The proposed system is divided into three stages, namely, preprocessing, machine learning, and output quality evaluation, which are next described.

#### Preprocessing Subsystem

In the preprocessing stage, the EGM and the corresponding pacing coordinates have to be available for designing a suitable supervised learning system. The EGM were retrieved by the implanted ICD during pace mapping in EPS, and pacing coordinates were simultaneously obtained by using a three-dimensional (3D) nonfluoroscopic CNS (Carto XP and Ensite NavX[11]). These systems can measure the catheters spatial positions inside the heart in real time, by means of electromagnetic fields generation and detection, and they can also build 3D electroanatomical maps for visualization purposes integrating temporal and spatial information of the EGM recorded from its set of catheters.

As described in [4, 5], EGM of two VTES can be distinguished as different when they are separated a distance (area) larger than 2 cm (8.9±9.0 cm^2^). For this reason, a LV division into eight regions, so-called *octants* for classification purposes, was manually done by a cardiac electrophysiology expert (JA) with recognized experience in ventricular mapping and use of CNS (see Fig 2 in [4]). The LV was divided by using three perpendicular planes, which separate the heart chamber into two halves for each plane, namely, apical vs basal, septal vs lateral, and inferior vs superior. The intersection of these planes generated a division into eight octants, namely: apical-septal-inferior (ASI), apical-septal-superior (ASS), apical-lateral-inferior (ALI), apical-lateral-superior (ALS), basal-septal-inferior (BSI), basal-septal-superior (BSS), basal-lateral-inferior (BLI), and basal-lateral-superior (BLS). In order to minimize the possible bias in the LV division, this process was blindly performed, with no *a priori* knowledge about the EGM at each pacing site. After LV division, CNS coordinates were assigned to one of the two halves for each plane, and to one of the eight octants. Regarding the regression scheme, CNS coordinates were dependent on the spatial reference system of each patient, and since the regressor design required consistent references for different patients, the mean vector was computed from the spatial coordinates of each patient, and subsequently subtracted from each patient location, thus setting the zero point as the reference for all patients.

Note that the VTES was known for the set of examples (also named instances) that are used for the machine design. However, when a new EGM is presented to the system in operation, the VTES is to be estimated instead, which is the aim of the proposed supported system.

#### Machine Learning Subsystem

**Fig 2:**
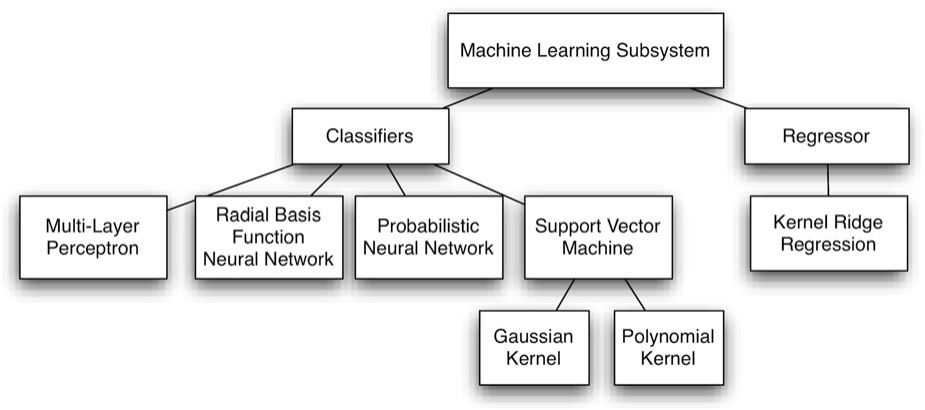
Diagram of the proposed machine learning subsystem.

The second stage of the proposed supporting system conveys a data model to determine the VTES (half or octant in classification, and octant coordinates in regression) as a function of the EGM. A supervised learning framework was followed for building a data model from the known VTES anatomical locations in a set of patients. Fig 2 shows the diagram of the proposed machine learning subsystem.

With respect to the machine learning classifiers, several classical and emergent classification schemes were benchmarked. Firstly, classical artificial neural networks were considered, such as Multi-Layer Perceptron (MLP) and Radial Basis Function Neural Network (RBFNN), for exploring a global and a local learning paradigm, respectively [10]. Secondly, Probabilistic Neural Networks (PNN) were also scrutinized, which are based on the theory of Bayesian classification and the estimation of probability density functions [12]. Finally, Support Vector Machines (SVM) were considered, since they offer good generalization ability by constructing classifiers with maximal margin between classes while minimizing the structural risk [13]. We next present a short overview of each classification scheme.

The MLP is a multilayer network, whose training is performed according to the back propagation error. The MLP training has two iterative phases: (a) forward phase, where the MLP parameters are fixed and the input is propagated through the network; (b) backward phase, where the error signal, obtained by comparing the MLP output and the desired target, is propagated through the network in the backward direction. In this second phase, adjustments are made to the network parameters [10]. The MLP network is often trained by using the conjugate gradient algorithm to minimize the sum-of-squared errors, while simultaneously finding the weights of the hidden and the output layers, as far as the input layer has no weights. Here, we considered just one hidden layer, apart from the input and the output layers, and the hidden and output activation functions were sigmoidal and linear, respectively.

The RBFNN is also a multilayer network with universal approximation capability, which has its origin in exact function interpolation [10]. Hidden and output activation functions are often Gaussian (radial basis function, RBF) and linear, respectively, and they can perform exact interpolation when a radial basis function is placed on every training instance (exact RBFNN). When instances are affected by noise (as it is usual in real-world data), the generalization ability can be improved by considering a regularization term to avoid over-fitting and to produce smoother solutions. The training algorithm for the exact RBFNN is much faster than that for MLP, since RBFNN parameters are not found by an iterative procedure, but instead applying the Moore-Penrose pseudoinverse.

The PNN is a variant of the previously described exact RBFNN, where the output layer has as many units as classes to discriminate [14]. Unlike the RBFNN, not all the hidden neurons of PNN are connected to all the output neurons, but instead, each hidden neuron is just connected to the output neuron associated to the class where the RBF is placed on. Thus, every output neuron provides with a discriminant function which can be seen as an estimation of the class probability density function. For classification, PNN assigns the instance to the class of the output neuron with the highest value.

The SVM is a statistical learning algorithm based on the Structural Risk Minimization principle [13, 15]. The SVM scheme maps the original input space to a high-dimensional space where classes are assumed to be linearly separable, so that it is possible to estimate a separating hyperplane maximizing the margin between the boundary and its closest instance. In SVM, a regularization parameter controls the trade-off between the training error and the margin size. The mapping is defined by means of a Mercer kernel function. In this work, a Gaussian kernel (GK) and a polynomial kernel (PK) have been considered for the nonlinear mapping.

With respect to the machine learning regressor, the actual spatial coordinates (*x*, *y*, *z*) of each pacing site were used as targets for regression. Since the spatial reference systems were patient-dependent, a common reference origin was established by setting it as the mean point of the available exit sites for each patient.

For the regression approach, nonlinear models with different input spaces and multidimensional outputs were built. The Kernel Ridge Regression (KRR) algorithm [16, 17], a nonlinear kernel version of the regularized least squares method, was considered given its capability for dealing with high dimensional data in a very computationally efficient way. A GK was also used in KRR in order to map the input space to a new feature space. KRR has been used in a wide field of applications such as, wind speed prediction [18], fMRI pattern prediction [19], face recognition [20] or estimating ink density from colour camera [21].

#### Output Quality Evaluation

A quality measurement stage was considered to provide with an idea of the uncertainty and statistical properties of the output provided by the classification and regression subsystems. The classifier output quality was evaluated in terms of the accuracy rate, using both binary and multiclass classifiers. Binary classifiers were used in order to determine one of two LV halves (there were three different binary classifiers). The multiclass classifier determined the octant where the LVTES was located. In addition, a different multiclass classifier combining the outputs provided by three binary classifiers was evaluated. The regressor output quality was assessed by computing the Euclidean distance between the actual and the estimated spatial coordinates, and this distance provided with the spatial resolution of the regressor system.

## Results

The proposed system was designed with the previously described database. For this purpose, different sets of experiments were conducted analyzing the performance and scope of the classification and regression machines, together with their comparison and benchmarking. The most relevant ones are reported next.

### Free Parameter Tuning

In order to design the machine learning classifiers (MLP, RBFNN, PNN, and SVM) and regressor (KRR), a wide enough range of values was explored for each free parameter, as follows:

- Number of hidden neurons in MLP: 10 values, from 1 to 10.
- Regularization parameter and Gaussian width for RBFNN: 10 equally spaced values in [10^−3^, 10^2^].
- Gaussian width in the PNN units: 10 equally spaced values in [0.1, 1].
- Regularization parameter, Gaussian width, and polynomial degree for SVM: 10 logarithmically equally spaced values in [10, 10^3^], 20 logarithmically equally spaced values in [10^−2^, 10], and ten integer values from 1 to 10, respectively.
- Regularization parameter and Gaussian width in KRR: 50 logarithmically spaced intervals were searched in the range from 10^−3^ to 10, and from $\log(0.1\hat{\sigma })$ to $\log(100\hat{\sigma })$, respectively, where $\hat{\sigma }$ is the mean distance between pairs of instances.

The most appropriate values for the free parameters were chosen according to a cross validation methodology [14] (5—fold cross validation in our experiments). In this methodology, a set of training instances are randomly partitioned into 5 subsets of the same size, so that one subset is reserved for evaluating the model constructed with the four remaining ones. This process is repeated 5 times, setting apart each of the 5 subsets just once. Results obtained with the 5 designs are averaged, and the values providing the best free parameters for each kind of classifier/regressor are selected. In our study, 10 realizations were run to obtain the average results from different cross validation partitions and initial weights in MLP.

In order to measure the performance generalization, a *leave one patient out* strategy was used to provide with a patient-independent result. This methodology is similar to N—fold cross validation, with N being the number of patients, this is: (1) data corresponding to one patient are set apart; (2) the machine is trained with data from the rest of patients; and (3) the performance is evaluated in the reserved patient. This procedure was repeated once for every patient in the database.

### Classification Performance

A first set of experiments was conducted with classification machines, aiming to determine the impact of the presence or absence of stimulation artifact (see Fig 1), the input space, and the classification algorithms. For this purpose, binary and multiclass classifiers were used to classify LV halves and octants, respectively. Table 2 shows the averaged accuracy rates of 10 realizations when using the far-field EGM waveform defined in dataset D1, both with and without stimulus, showing that similar performance was in general obtained. Hence, it can be concluded that no noticeable distorting effect was introduced in the classification analysis due to the pace mapping stimulus.

**Table 2:** Average accuracy rate (in %) for the classifiers using dataset D1.

		Halves			Octants	
Waveform	Classifier	Apical / Basal	Septal / Lateral	Superior / Inferior	Binary combination	Multiclass
Far-field EGM with stimulus	MLP	53.0	70.6	62.1	23.0	22.1
	RBFN	51.4	70.8	56.0	20.2	20.3
	PNN	53.0	63.6	55.7	19.0	18.8
	SVM-GK	49.9	73.5	54.2	20.2	20.5
Far-field EGM without stimulus	MLP	52.9	68.3	57.7	19.7	22.0
	RBFN	49.5	70.2	53.9	19.3	19.1
	PNN	56.4	63.8	55.4	20.2	19.3
	SVM-GK	52.3	73.0	52.0	18.8	22.9
Bipolar EGM with stimulus	MLP	56.0	54.3	48.4	12.9	11.9
	RBFN	57.2	55.7	53.0	16.1	14.6
	PNN	58.4	56.0	56.4	14.8	21.9
	SVM-GK	56.0	53.5	51.6	14.8	14.8
Far-field & Bipolar EGM with stimulus	MLP	55.2	69.1	64.5	23.4	19.0
	RBFN	63.7	69.1	58.9	24.8	24.6
	PNN	54.7	65.0	52.3	19.0	21.9
	SVM-GK	60.3	71.3	57.2	23.8	25.5

Table 2 also shows that multiclass and binary classifiers have some discriminant capabilities with both far-field and bipolar EGM waveform input spaces. Apart from apical/basal, far-field EGM waveform has more discriminant capabilities than bipolar EGM waveform, and in general, the best performance with dataset D1 was achieved by using the concatenation of far-field and bipolar EGM waveforms. Discrimination between septal/lateral half got the best performance using the SVM classifier with GK (SVM-GK) and far-field EGM waveform as input space, whereas poor performance was obtained for apical/basal and superior/inferior halves in binary classification. Note at this point that the averaged accuracy rate for random choice among 8 classes (octants) is 12.5%, hence octant classification was significantly improved with the use of machine learning techniques, with 25.5% of accuracy for multiclass SVM-GK classifier and combining far-field and bipolar EGM waveforms.

Table 3 shows the averaged accuracy rates of 10 realizations by using dataset D2 (input spaces given by time and voltage EGM features). In this case, the SVM classifier with PK (SVM-PK) was also considered due to the good performance of this kind of kernel when dealing with low-dimensional input spaces. Octant classification with binary classifiers obtained the highest performance, 34.9% and 31.8%, for SVM-GK and input spaces 2*VR*1*T* and 3*V*1*T*, respectively. With respect to halves classification, apical/basal accuracy increased significantly with respect to the use of EGM waveform (71.3% with 2*VR*1*T* and SVM-GK), while similar discriminant capabilities were obtained in septal/lateral (with 3*V*1*T* and 3*V* input spaces, SVM-GK) and in superior/inferior (with 2*VR*1*T*, SVM-GK). Note in bold the best results for datasets D1 and D2 in Table 2 and Table 3 for each input space. In spite of the imbalance in the number of pacing sites among octants, the best performance was checked not to correspond to the octant conveying a larger number of EGM.

**Table 3:** Average accuracy rate (in %) for the classifiers using dataset D2.

		Halves			Octants	
Features	Classifier	Apical / Basal	Septal / Lateral	Superior / Inferior	Binary combination	Multiclass
3*V*	MLP	55.9	74.3	58.3	22.5	21.0
	RBFN	58.1	75.2	59.1	23.1	22.9
	PNN	56.6	72.8	61.9	23.1	25.3
	SVM-GK	57.1	**75.4**	58.1	24.6	27.0
	SVM-PK	50.1	74.5	58.5	20.5	24.1
3*V* 1*T*	MLP	67.2	73.8	56.5	28.0	26.4
	RBFN	69.8	74.1	61.7	31.8	**31.6**
	PNN	68.4	73.0	61.2	27.7	27.7
	SVM-GK	67.7	**75.4**	60.0	31.8	27.9
	SVM-PK	64.6	74.4	57.8	29.1	28.4
3*V* 3*T*	MLP	55.6	73.3	59.8	23.5	23.1
	RBFN	55.5	73.1	61.7	24.4	24.1
	PNN	56.9	71.8	57.1	19.8	21.9
	SVM-GK	49.9	73.0	56.4	19.5	20.0
	SVM-PK	54.0	74.7	56.9	23.4	26.0
2*V R*1*T*	MLP	67.4	70.5	61.5	28.9	29.1
	RBFN	69.7	71.7	61.5	28.5	29.4
	PNN	64.6	72.5	60.7	26.5	26.5
	SVM-GK	**71.3**	71.3	**65.3**	**34.9**	26.3
	SVM-PK	66.0	70.8	58.1	26.7	24.1

In light of the outcome, in general terms it follows that features 2*VR*1*T* and 3*V*1*T* yield the best performance in octant and halves classification. Interestingly, in spite of more electrical information being present in the waveform, no better performance was obtained when using it, probably because of the effect of the high dimensionality of the input space. Besides, SVM-GK usually works slightly better than neural networks classifiers.

Previous results were obtained by accounting the pacing sites placed on the scar or on normal tissue. Table 4 shows the averaged accuracy rates of 10 realizations by using SVM-GK (the best classifier previously obtained) and considering separately the type of tissue when pacing, namely, scar (bipolar peak-peak voltage was < 1.5 mV), normal (bipolar peak-peak voltage was ≥ 1.5 mV), and both. The highest accuracies for each type of tissue are in bold. In general, the best performance was obtained when considering both normal and scar tissue. However, these results could be due to the machine learning nature of our system, which is able to obtain better generalization ability with a more representative design set (in general, with larger number of examples). In addition, it can be checked that septal/lateral classifier provided higher accuracy rate in normal than in scar tissue, whereas the opposite is true for superior/inferior classification. Comparing the results in Table 4 with those in Table 2 and Table 3, we may conclude that input spaces 3V1T and 2RV1T provided the best performance when considering scar+normal tissues. They also yielded the best accuracy when considering normal and scar+normal tissues, both for halves and octants classification. Better performance was obtained with 3V3T for scar tissue in septal/lateral and superior/inferior halves for multiclass classification.

**Table 4:** Average accuracy rate (in %) for the SVM-GK classifier using dataset D1 and D2 when considering pacing in scar, normal or both tissue.

		Halves			Octants	
Tissue	Input space	Apical / Basal	Septal / Lateral	Superior / Inferior	Binary combination	Multiclass
Scar	3V	47.1	65.7	60.8	23.5	26.5
	3V1T	**63.7**	59.8	61.8	25.5	26.5
	3V3T	49.0	**66.7**	**65.7**	22.5	**27.4**
	2VR1T	61.8	59.8	62.7	**26.5**	25.5
	Far-field EGM waveform	33.3	45.1	52.9	10.8	18.6
	Far-field & bipolar EGM waveform	27.4	38.2	61.8	7.8	21.6
Normal	3V	42.0	80.2	46.9	13.6	9.8
	3V1T	**60.5**	77.8	43.2	**23.5**	16.0
	3V3T	51.8	**82.7**	44.4	17.3	27.2
	2VR1T	45.7	81.5	**61.7**	19.7	**32.1**
	Far-field EGM waveform	37.0	65.4	51.8	16.0	14.8
	Far-field & bipolar EGM waveform	51.8	74.1	42.0	17.3	13.6
Both	3V	47.0	74.9	53.5	17.5	28.4
	3V1T	60.1	**80.3**	51.4	26.8	26.2
	3V3T	52.4	77.0	54.6	15.8	23.5
	2VR1T	58.5	78.7	**66.1**	**35.0**	**30.6**
	Far-field EGM waveform	**61.7**	75.4	51.9	22.9	22.9
	Far-field & bipolar EGM waveform	49.2	63.9	56.3	15.3	22.4

### Regression Performance

The usefulness of machine learning for regression was benchmarked in terms of octant regression performance. The spatial coordinates of LVT pacing sites were estimated by using KRR and datasets D1 and D2. The spatial resolution was measured as the Euclidean distance between the actual and the estimated pacing spatial coordinates.

Table 5 shows the Euclidean distance (mean and standard deviation) between the actual and the estimated spatial coordinates for 138 pacing sites in datasets D1 and D2 when pacing in scar, normal, and both tissues. Slightly larger distances were obtained for normal tissue in comparison with scar tissue. A Wilcoxon rank sum test, with significance level at *α* = 0.05 [22], was performed for statistical comparison of: (1) type of tissue, obtaining significant differences for normal + scar (both) vs normal, and normal vs scar pacing sites; and (2) input space, with significant differences for 2*VR*1*T* vs the rest of input spaces.

**Table 5:** Euclidean distance (mean and standard deviation, in cm) between the actual and estimated spatial coordinates for pacing sites in datasets D1 and D2. Scar, normal and both (normal and scar) tissues were considered.

	Pacing sites		
Input spaces	Both	Scar	Normal
**3V**	3.10 ± 1.32	3.09 ± 1.29	3.77 ± 1.43
**3V1T**	2.99 ± 1.31	2.95 ± 1.16	3.80 ± 1.37
**3V3T**	3.10 ± 1.34	2.78 ± 1.04	3.83 ± 1.52
**2VR1T**	2.84 ± 1.28	2.71 ± 1.03	3.55 ± 1.32
**Far-field EGM waveform**	3.12 ± 1.34	3.09 ± 1.18	4.03 ± 1.44
**Bipolar EGM waveform**	3.19 ± 1.24	3.18 ± 0.99	3.70 ± 1.44
**Far-field—bipolar EGM waveform**	2.99 ± 1.26	3.04 ± 0.96	3.87 ± 1.38

Distances in Table 5 are in agreement with the spatial resolution reported by other clinical works in the literature. Recall that those works had reported a mean spatial resolution of 8.9±9 cm^2^ (range from 0 to 35 cm^2^) [5] and a distance ≥ 2 cm to distinguish EGM from two pacing sites [4].

## Discussion

An automatic system has been proposed, tested its general feasibility, and technically evaluated in the present work for supporting the anatomical region identification of the LVTES in EPS, from the analysis of ICD EGM. The system consists of a preprocessing stage, a machine learning subsystem for classification/regression, and the statistical quality characterization of the locations provided by the machine. The proposed system has been analyzed using a database of EGM registered by ICD during a pace mapping protocol and simultaneously storing the pacing electrode coordinates in a CNS, hence providing a data-driven model for the LVTES identification. EGM in this database were registered with the ICD electrode in the right ventricle, while pacing was applied at different LV locations from a different exploring catheter. Previous studies [4–6] have reported a spatial resolution of several cm, hence the anatomical region identification for the LVTES was tackled by dividing the LV in anatomical regions (halves and octants) based on clinical criteria. This approach allowed us to address the automatic anatomical region identification from a classification point of view. In addition, pacing coordinates were used to obtain the spatial resolution of our system and compare its performance with that obtained in the clinical practice. For this purpose, a regression approach was followed.

Simultaneous consideration of classification and regression learning methods provided consistent and complementary information. In both, input spaces given by simple EGM features (time intervals and voltages) yielded better performance than the raw EGM waveform. With respect to the classification machines, the SVM yielded in general slightly better performance than other benchmarked neural networks. The best discriminant capability was obtained for septal/lateral classifier when pacing was applied in just normal and in normal+scar tissues, whereas superior/inferior halves were better classifier in scar tissue. With respect to the spatial resolution, similar results were obtained for both features and waveform input spaces. Slightly better resolution was obtained in scar and scar+normal than normal tissue. Spatial resolution provided by our automatic system was in agreement with previous clinical studies [4–6].

Several limitations can be pointed out in the present work. Although the number of patients is remarkable for this kind of database, more representativeness could be obtained from a multicenter study. In addition, several octants could not be well identified using just the information provided by the EGM stored in ICD, and additional covariates are required for increased accuracy in this setting. From a theoretical point of view, signals such as the ECG or intracardiac sonographic recordings could provide relevant covariates, however, their use in ICD patient populations is limited. Finally, the classification and regression performance can seem low when compared to other machine learning applications. However, one should keep in mind that our proposal focuses on an automatic system, which can bring into the clinical practice a tool for supporting the EPS for arrhythmia ablation. In addittion, the apparently moderately-high accuracy in this application can bring relevant advantages, such as time reduction of EPS duration.

In spite of previous evident limitations, we can conclude that the proposed system allows to support the anatomical region identification of the LVTES in ablation procedures whose EGM has been stored in ICD. Besides, the proposed system and methodology can be useful to drive the design of new machines with improved performance.
